# Adductor canal block with periarticular infiltration versus periarticular infiltration alone after total knee arthroplasty: A randomized controlled trial protocol

**DOI:** 10.1097/MD.0000000000020213

**Published:** 2020-05-15

**Authors:** Yongcheng Ren, Jiacai Liao, Xiaoyan Qin, Jianming Yang

**Affiliations:** a Department of Anesthesiology; b Department of Ophthalmology and Otorhinolaryngology, Qianjiang District Chinese medicine hospital of Chongqing, Chongqing, China.

**Keywords:** adductor canal block, periarticular infiltration, study protocol, total knee arthroplasty

## Abstract

**Background:**

Effective postoperative analgesia may enhance early rehabilitation after total knee arthroplasty (TKA). The purpose of this study is to perform a randomized controlled trial to compare the efficiency of adductor canal block (ACB) with periarticular infiltration (PAI) versus PAI alone for early postoperative pain treatment after TKA.

**Methods:**

After institutional review board approval, written informed consent was obtained from patients undergoing elective TKA. Subjects were randomized into 2 groups as follows: adductor canal blockade with 30 mL of 0.5% ropivacaine and 100 mcg of clonidine. All patients received a periarticular infiltration mixture intraoperatively with scheduled and patient requested oral and IV analgesics postoperatively for breakthrough pain. The primary outcome was morphine consumption in the first 24 hours. Secondary outcomes included pain scores, morphine consumption at 48 hours, opioid-related side effects (post-operative nausea/vomiting, sedation scores), functional outcomes, quadriceps strength, and length of hospital stay.

**Conclusions:**

For the present trial, we hypothesized that patients receiving adductor canal block + PAI would have significantly lower morphine consumption and pain scores after surgery.

**Trial registration number:**

researchregistry5490

## 1 Introduction

Pain management after total knee arthroplasty (TKA) continues to evolve especially as early postoperative analgesia is of paramount importance in achieving patient satisfaction and improved clinical outcome. The goal of achieving an ideal analgesia modality which facilitates early rehabilitation, prevents knee stiffness, reduces hospital stay due to pain, and promotes good functional outcomes continues to be elusive. Multimodal analgesia has been devised to control postoperative pain and reduce opioid consumption including periarticular injection (PAI) and peripheral nerve blocks.^[1,2,3]^

Femoral nerve block has been one of the most commonly used peripheral nerve blocks following TKA. However, because the femoral nerve is comprised of both sensory and motor branches, this block can lead to quadriceps weakness thus delaying mobilization and presenting an increased risk of falls.^[4,5]^ The adductor canal block (ACB) has recently gained popularity as an alternative to femoral nerve block due to reduced incidence of quadriceps muscle weakness. It provides a more distal nerve blockade, at the mid-thigh, ideally providing sensory blockade in the distribution of the saphenous nerve, posterior branch of the obturator nerve and vastus medialis nerve while sparing quadriceps function.^[6]^ Literature has supported improved motor function with similar pain control when comparing ACB to femoral nerve block.^[7,8,9,10]^

The PAI is a surgeon-controlled analgesic technique that used to reduce the pain in the early postoperative period with no influence on quadriceps strength. It is a mixture of medications that typically includes, but is not limited to, a local anesthetic, morphine, and Toradol. It has been shown to have superior analgesic effects compared to placebo^[11,12]^ and non-inferior compared to femoral nerve block.^[13,14,15,16]^ PAI during TKA has thus been recommended for routine application considering effective pain relief and smoother rehabilitation.

Despite the evidence above, concerns exist regarding the incomplete analgesia of PAI. Previous studies have been published to explore the efficacy between ACB + PAI versus PAI alone in reducing pain after TKA, but with different conclusions.^[17,18,19,20]^ We, therefore, further designed a randomized controlled study to compare ACB + PAI with PAI in the treatment of TKA. For the present trial, we hypothesized that the addition of an ACB to PAI, compared with PAI alone, would reduce the time to meet discharge criteria after total knee replacement. Additionally, we hypothesized that patients receiving ACB + PAI would have significantly lower pain scores after surgery.

## 2 Material and method

### 2.1 Study design and patient enrollment

This prospective, blinded randomized controlled trial was conducted at our single university hospital. The study was approved by the institutional review board of the hospital (CQ001023). The trial was registered with Research Registry (researchregistry5490) prior to the enrollment start. Patients were enrolled after signed written consent was obtained. All surgeons, recovery room and floor nurses, research assistants, statisticians, and patients were blinded to group allocation. Only the anesthesiologists performing the blocks and operating room nurses were not blinded.

Patients aged 50-80 years with a body mass index of 18-36 kg/m^2^ and an American Society of Anesthesiologists functional status of I-III were included. Exclusion criteria included a knee flexion deformity of ≥30°, varus-valgus deformity of ≥30°, a diagnosis of nonosteoarthritis (including rheumatic arthritis, traumatic arthritis, and septic arthritis), known allergy to the drugs used in this study, or a past history of opioid consumption, excessive alcohol consumption, cognitive impairment, psychiatric illness, narcotic dependency, recognized neuromuscular disorder, knee surgery (including arthroscopy and open surgery), knee infection, or thrombolytic events (including myocardial infarction, cerebrovascular accident, deep vein thrombosis, and pulmonary embolus). Patients who had an inability to communicate verbally or who were unwilling to give informed consent were also excluded.

### 2.2 Randomization

**Figure 1:**
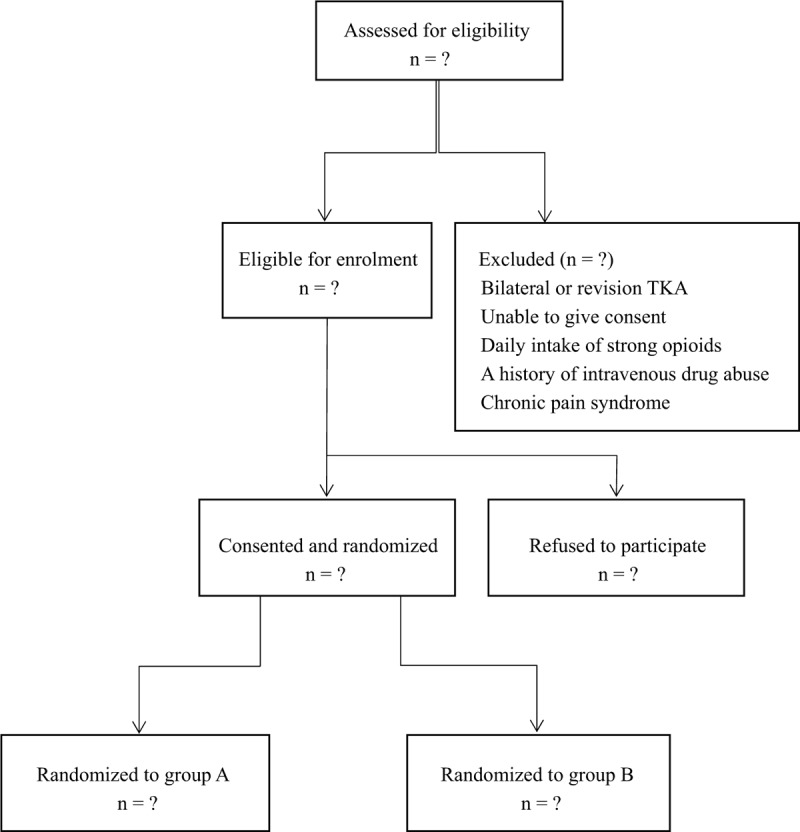
Consolidated Standards of Reporting Trials Statement flow diagram.

Randomization was done by a secretary using a computer-generated randomization list (Research randomizer, www.randomizer.org) in a 1:1 ratio with 20 numbers in each block. Every participant received a consecutive study number from 1 to 69 and received the treatment assigned according to the randomization list. The list was stored and only 2 nurses, who prepared the study medications, had access to it. They had no interactions with the patients. All other clinical personnel, participants and outcome assessors were blinded to the intervention. The randomization key was first broken when all enrolled patients had completed the study. After discharge, the participant's personal information was eliminated from the study number and was therefore not traceable back to the patient (Fig. 1).

### 2.3 Intraoperative interventions

Both groups received preoperative oral meloxicam (15  or 7.5 mg if the patient was > 74 years of age) and extended-release oxycodone (10 mg). Both groups received a spinal anesthetic with 0.5% bupivacaine (10 or 12.5 mg). Patients received 4 mg of ondansetron and 20 mg of famotidine. Patients were not given intravenous opioids or ketamine. The surgeons administered a deep periarticular injection prior to cementation and a second superficial injection prior to closure. The deep injection consisted of 30 mL of 0.5% bupivacaine with epinephrine, 1 mL of 8 mg/mL morphine, 1 mL of 40 mg/mL methylprednisolone, 500 mg of cefazolin in 10 mL, and 22 mL of normal saline solution. The superficial injection was 20 mL of 0.25% bupivacaine.

For ACB, an ultrasound transducer was used to identify the adductor canal. The transducer located the adductor canal at mid-thigh, halfway between the inguinal crease and patella. Superficial femoral artery, sartorius muscle, adductor longus muscle, and adductor magnus muscle were identified. The hyper echoic structure located anterolateral to the artery (saphenous nerve and nerve to vastus medialis) was identified as the target injection site. A 22-guage, 100 mm needle was introduced lateral to medial under ultrasound guidance using linear probe of a sonosite machine. Solution containing 30 mL of 0.5% ropivacaine and 100 mcg of clonidine was injected after ensuring correct placement of the needle.

### 2.4 Postoperative interventions

Patients in both groups received 1 session of physical therapy on the day of the surgical procedure and 3 sessions at 24 and 48 hours until they achieved discharge criteria. Patients defaulted to 2 physical therapy sessions if they did not meet discharge criteria by 72 hours. Each rehabilitation session assessed patients for discharge. The criteria for discharge were the ability to independently transfer in and out of a bed, chair, or toilet seat; to walk approximately 50 m with or without an assistive device; to negotiate stairs with or without a rail or cane; and to perform a home exercise plan. All subjects underwent a standard postoperative multimodal pain management regimen. Postoperative medications included acetaminophen, ketorolac followed by celecoxib (for 3 months), gabapentin (standing order for 10 days), oral opioids (as needed), and intravenous hydromorphone for breakthrough pain.

### 2.5 Outcomes and measures

The primary end point was Morphine consumption in the first 24 hours (including Morphine administered intra-operatively and in recovery). Secondary outcomes included pain scores, Morphine consumption at 48 hours, opioid-related side effects (post-operative nausea/vomiting, sedation scores), functional outcomes, quadriceps strength, and length of hospital stay. Pain scores were recorded at 1, 6, 12, 24, and 48 hours post-operatively, using a Numerical Rating Scale (0–10) at rest and during 45° passive flexion of knee.

Nausea was defined as the unpleasant sensation associated with awareness of the urge to vomit; vomiting was defined as the forceful expulsion of gastric contents from the mouth. The presence/absence of nausea and the number of episodes of vomiting (more than 10 mL) were recorded. The usage of antiemetics was also recorded. The level of sedation was assessed on a 4-point scale (0 = no sedation, 1 = light, 2 = moderate, 3 = severe).

Functional outcome measures used were the 30 Seconds Chair Stand Test (30-CST) and the Timed Up and Go (TUG) test. 30-CST tested the number of times each subject was able to stand up from a seated position on a chair in 30s. The TUG test assessed the time taken for each subject to get up from a seated position on the chair, walk 3 meters away from the chair before returning to it. Both 30-CST and TUG test had been shown to be reliable and validated tools for rehabilitation of orthopedic patients. Quadriceps strength was assessed as a percentage of the baseline measurements at 24 and 48 hours post-operatively, using the method previously described. The 30-CST and TUG tests were performed at 24 and 48 hours. We also assessed for early complications such as hematoma, infection and neurological deficits at 48 hours. Any other early complications, including falls within the first 48 hours were recorded. The length of hospital stay was also noted.

### 2.6 Sample size calculation

The sample size calculation was based on a pilot study that we conducted on 18 patients (whose data were not included in the present study). In this prior study, the mean difference and standard deviation of the Numerical Rating Scale scores 24 hours after the operation between the ACB + PAI and PAI groups were 0.40 and 0.19, respectively. From this, it was determined that 42 subjects would be required to reach an α value of 0.05 and a power of 85%. It was estimated that the attrition rate due to canceled surgery or reasons of late patient ineligibility could be up to 20% and, therefore, to account for this, the final sample size selected was n = 100 (50 per group).

### 2.7 Statistical analysis

The statistical analyses in this study were performed using the Statistical Package for the Social Sciences (SPSS (IBM, USA)) 20.0 software. Continuous variables were presented in the form of mean ± standard deviation or error. The Kolmogorov–Smirnov normality test was used to assess continuous variables. Group comparisons on the variables that showed normal distribution were performed using one-way analysis of variance. Mann–Whitney *U* variance analysis was used for discrete numerical variables that did not show normal distribution. Relationships between the categorical variables were determined by preparing crosstabs and using the Chi-squared (χ^2^) test. *P* < .05 was accepted as statistically significant.

## 3 Discussion

TKA is a common surgical procedure that can cause severe postoperative pain. Various methods for postoperative analgesia management are available, such as systemic opioids, epidural local anesthetic, peripheral nerve block, and local anesthetic infiltration analgesia.^[2,3,4,5]^ Use of systemic opioids can cause adverse effects that may affect functional rehabilitation, such as nausea, vomiting, pruritus, sedation, and respiratory depression. Hypotension, urinary retention, and pruritus are more common in patients with epidural analgesia.^[8]^

The introduction of ACB to manage pain after TKA is relatively new. The adductor canal is an aponeurotic space in the middle third of the thigh. It contains nerve branches that supply sensory innervation to the knee, including the posterior branch of the obturator nerve and the saphenous nerve. Blocking these nerves provides analgesia to the medial aspect but not to the lateral or posterior aspects, of the knee.^[11,21]^ Moreover, PAI has been shown to improve pain management and to preserve motor function. With PAI, the nerves, muscle, and tissue in the posterior, lateral, and medial aspects of the knee are infiltrated intraoperatively with local anesthetic, morphine, and ketorolac to provide analgesia.^[22]^

The main limitation of the current study was the inability to blind both the participants and the physicians to comparisons between peripheral nerve blockade and periarticular injection. This lack of blindness may have introduced some risk of bias from both the patients and the physicians. The outcome assessments from the adjudicators and all the statistical analyses were conducted in a blinded manner. In addition, the impossibility of measuring quadriceps muscle power before and after the operation using special instruments was another limitation. If this had been possible, the evaluation between the ACB + PAI and the PAI groups could have been more objective.

## Author contributions

**Conceptualization:** Yongcheng Ren.

**Data curation:** Yongcheng Ren.

**Formal analysis:** Yongcheng Ren.

**Funding acquisition:** Jianming Yang.

**Investigation:** Xiaoyan Qin, Jiacai Liao.

**Methodology:** Xiaoyan Qin, Jiacai Liao.

**Resources:** Jianming Yang.

**Software:** Xiaoyan Qin.

**Supervision:** Jianming Yang.

**Validation:** Xiaoyan Qin.

**Visualization:** Jianming Yang.

**Writing – original draft:** Yongcheng Ren.

**Writing – review & editing:** Yongcheng Ren, Jianming Yang.
